# Characteristics associated with uncomplicated pregnancies in women with obesity: a population-based cohort study

**DOI:** 10.1186/s12884-021-03663-2

**Published:** 2021-03-05

**Authors:** Sophie Relph, Yanfang Guo, Alysha L. J. Harvey, Matias C. Vieira, Daniel J. Corsi, Laura M. Gaudet, Dharmintra Pasupathy

**Affiliations:** 1 Department of Women and Children’s Health, King’s College London, 10th Floor, St Thomas’ Hospital, Westminster Bridge Road, London, UK; 2 Better Outcomes Registry & Network Ontario, Children’s Hospital of Eastern Ontario, Ottawa, Canada; 3 OMNI Research Group, Clinical Epidemiology Program, Ottawa Hospital Research Institute, Ottawa, Canada; 4 Children’s Hospital of Eastern Ontario Research Institute, Ottawa, Canada; 5 School of Epidemiology and Public Health, University of Ottawa, Ottawa, Canada; 6 Department of Obstetrics and Gynaecology, University of Campinas (UNICAMP), School of Medical Sciences, 101 Alexander Fleming st, Cidade Universitaria, Campinas, SP Brazil; 7 Department of Obstetrics and Gynecology, University of Ottawa, Ottawa, Canada; 8 Department of Obstetrics and Gynaecology, Queen’s University, Kingston, Ontario Canada; 9 Discipline of Obstetrics, Gynaecology and Neonatology, Westmead Clinical School, Faculty of Medicine and Health, University of Sydney, Camperdown, Australia

**Keywords:** Pregnancy, Antenatal, Uncomplicated, Obesity, Low-risk, Maternity, Cohort study, Registry

## Abstract

**Background:**

Approximately one in five pregnant women have obesity. Obesity is associated with an increased risk of antenatal, intrapartum, and perinatal complications, but many women with obesity have uncomplicated pregnancies. At a time where maternity services are advocating for women to make informed choices, knowledge of the chance of having an uncomplicated (healthy) pregnancy is essential. The objective of this study was to calculate the rate of uncomplicated pregnancy in women with obesity and evaluate factors associated with this outcome.

**Methods:**

This prospective cohort study was conducted using the Ontario birth registry dataset in Canada (703,115 women, April 2012–March 2017). The rate of uncomplicated or complicated composite pregnancy outcomes (hypertensive disorders of pregnancy, gestational diabetes, preterm birth, neonate small- or large- for gestational age at birth, congenital anomaly, fetal death, antepartum bleeding or preterm prelabour membrane rupture) were calculated for women with and without obesity. Associations between uncomplicated pregnancy and maternal characteristics were explored in a population of women with obesity but without other pre-existing co-morbidities (e.g., essential hypertension) or obstetric risks identified in the first trimester (e.g., multiple pregnancy), using log binomial regression analysis.

**Results:**

Of the studied Ontario maternity population (body mass index not missing) 17·7% (*n* = 117,236) were obese. Of these 20·6% had pre-existing co-morbidities or early obstetric complicating factors. Amongst women with obesity but without early complicating factors, 58·2% (*n* = 54,191) experienced pregnancy without complication; this is in comparison to 72·7% of women of healthy weight and no early complicating factors. Women with obesity and no early pregnancy complicating factors are more likely to have an uncomplicated pregnancy if they are multiparous, younger, more affluent, of White or Black ethnicity, of lower weight, with normal placental-associated plasma protein-A and/or spontaneously conceived pregnancies.

**Conclusions:**

The study demonstrates that over half of women with obesity but no other pre-existing medical or early obstetric complicating factors, proceed through pregnancy without adverse obstetric complication. Care in lower-risk settings can be considered as their outcomes appear similar to those reported for low-risk nulliparous women. Further research and predictive tools are needed to inform stratification of women with obesity.

**Supplementary Information:**

The online version contains supplementary material available at 10.1186/s12884-021-03663-2.

## Background

Worldwide, the prevalence of obesity (body mass index (BMI) ≥30 kg/m^2^) in women of reproductive age is rising, affecting approximately 31.8% of pregnant women in the United States of America, 21.3% in the United Kingdom (UK) and 19.0% in Canada [1–3]. Whilst it is well documented that women with obesity have at least a two-fold increase in prenatal complications (gestational diabetes, hypertensive disorders, thromboembolism) [4], it is unclear what proportion of women may have uncomplicated pregnancies, with previous studies either targeted at the absence of a previous complication, or reporting on uncomplicated pregnancy and birth together.

Two studies have been identified which report on prevalence of uncomplicated pregnancy for women with obesity. The UK Birthplace study (2014) reported that only 12.9% of women with obesity had a medical risk factor (e.g. asthma, diabetes) at the onset of pregnancy and by 37 weeks’ gestation, less than half (45.4%) had either a pre-existing or new obstetric risk factor (e.g. previous Caesarean section, pre-eclampsia) [5]. The authors reporting a secondary analysis of a prospective Dutch cohort study (2014) compared obstetric referral rates for pregnant women with obesity, to those for women of healthy weight. They reported no difference in the rate of obstetric referral between women of normal BMI (18.5–24.9 kg/m^2^) and BMI 30–35 kg/m^2^. Of women with a BMI of 35 kg/m^2^ or above, 55% remained with midwifery-led care throughout pregnancy, and 30% had births in midwifery-led settings. Multiparity was associated with a greater chance of a midwifery-led pregnancy and birth across all BMI groups [6]. Vieira et al. (2017) studied the rate of uncomplicated pregnancy with uncomplicated birth, finding that 36.0% of women with obesity from their small cohort (*n* = 1409) had uncomplicated pregnancies and births. This outcome could be predicted using a combination of early pregnancy factors (multiparity, plasma adiponectin, glycated haemoglobin, maternal age and systolic blood pressure) with an AUROC of 0.72 (95% CI 0.68 to 0.76) [7].

The Ontario Public Health Association document on Informed Decision Making for Labour and Birth recognises that ‘In order to make an informed decision, individuals must receive the best scientific evidence of the benefits, risks and alternatives of an intervention from their [healthcare professional]’ [8]. Other settings, such as the UK, are also advocating for personalised care in maternity, where women are empowered to make informed choices [9]. A qualitative evidence synthesis which studied how women with obesity perceive prenatal and birth risks and make related choices identified that women’s relationships with their healthcare professionals and the nature of counselling received were influential in both [10]. Understanding the prevalence of uncomplicated pregnancy and its related factors, together with accurate prognostic information, is required to implement this vision of promoting evidence-based choices, both for pregnancy and for birth.

## Methods

This study has been reported according to the guidelines issued in the STROBE statement (STrengthening the Reporting of OBservational studies in Epidemiology). This is available in Additional file 1.

### Aim

The aim of this study was to calculate the rate of an uncomplicated pregnancy (antenatal period) in pregnant women with obesity and to determine the demographic and clinical factors which are associated with such uncomplicated pregnancies. We also set out to determine if the prediction of uncomplicated pregnancy using identified factors is feasible amongst women with obesity.

### Study design

This population-based cohort study was conducted using routinely collected data stored within BORN (Better Outcomes Registry and Network) Ontario. BORN is a prescribed maternal, newborn and child registry funded by the Ontario Ministry of Health and Long-Term Care. During the study period (April 2012 – March 2017), the data was entered from the pregnancies and births of 703,115 women.

### Study population

Women with pregnancies ongoing beyond 22 + 0 gestational weeks, with complete data on weight and the exposures of interest (complete case analysis) were included. Women were included regardless of BMI, because a comparison was conducted between women of healthy and obese weights. Subgroups were formed through identification of early pregnancy complicating factors, defined as either pre-existing medical comorbidities or obstetric complicating factors present in the first trimester (Table 1). Otherwise healthy women are considered so because they do not have co-morbidities or early pregnancy complicating factors.

**Table 1:** Factors used to define the early pregnancy complicating factors and uncomplicated pregnancy composite measures

**Early pregnancy complicating factors**	
***Pre-existing medical co-morbidities:***	• Autoimmune conditions e.g., lupus • Cardiovascular conditions e.g., congenital or acquired heart disease, pre-existing hypertension • Type 1 or 2 diabetes • Endocrine e.g., hyperthyroidism • Gastrointestinal e.g., inflammatory bowel disease, hepatitis • Renal disease • Haematology e.g., sickle cell disease, thrombophilia • Neurology e.g., epilepsy • Pulmonary e.g., cystic fibrosis, previous pulmonary embolism • Psychiatric e.g., Bipolar disorder, schizophrenia • Infectious disease e.g., active tuberculosis, human immunodeficiency virus
***Early obstetric complicating factors:***	• Alcohol/illicit drug exposure in pregnancy • Multiple pregnancy • Previous preterm birth • Previous stillbirth
**New onset obstetric complications**	
	• Gestational hypertension/pre-eclampsia/eclampsia • Gestational diabetes • Fetal anomaly • Intrauterine fetal death (> 20 + 0 weeks gestation or > 500 g at birth) • Small-for-gestational age (birthweight <10th centile) • Intrauterine growth restriction (birthweight <3rd centile) • Large-for-gestational age (birthweight >90th centile) • Placental abruption • Persistent and unexplained antepartum vaginal bleeding • Preterm birth • Premature rupture of membranes

### Exposures and outcomes

Pre-pregnancy BMI was calculated (kg/m^2^) using maternal pre-pregnancy weight and height. Weight was estimated from two sources: pre-pregnancy weight calculated by subtracting 2 kg from the first trimester weight recorded in the Perinatal Screening database in BORN Ontario, or self-reported pre-pregnancy weight recorded in the aggregated BORN Ontario dataset. Weight recorded in the Perinatal Screening database was prioritised because this was primarily measured rather than self-reported. The method of subtracting 2 kg to calculate pre-pregnancy weight from first trimester weight was established following modelling by Cheikh Ismail et al. (2016) [11]. Height was taken from that self-reported in the aggregated pregnancy dataset.

The primary outcome was the rate of uncomplicated pregnancy (antenatal period) in women with obesity. Uncomplicated pregnancy is a composite measure (Table 1) defined by the absence of both pre-existing medical co-morbidities (e.g., type 2 diabetes, essential hypertension), pre-existing/early obstetric (e.g., multiple pregnancy) or new-onset obstetric (e.g., gestational diabetes, pre-eclampsia) complications. Uncomplicated labour and birth amongst the same group of women is outside the scope of this analysis, because its prediction is most suited to women in whom alternative low risk birthplace choices could most safely be offered, i.e., those women who have already had an uncomplicated pregnancy. It is therefore planned as a follow-up analysis.

We planned to compare the rate of uncomplicated pregnancy (absence of new onset pregnancy complications) (Table 1) between otherwise healthy women (no pre-existing medical or early obstetric complicating factors) with BMI above or below 30 kg/m^2^, and between women with a BMI in the obese range but with or without pre-existing medical or early obstetric complicating factors.

Finally, we planned to examine sociodemographic and clinical factors and determine whether they were associated with uncomplicated pregnancy in otherwise healthy women with obesity. The planned sociodemographic and clinical factors for study were based on a priori hypothesis and availability of data items: maternal age at conception, pre-pregnancy BMI (reported as continuous, not categorical), parity, race, first language (native/non-native), neighbourhood income quintiles, smoking or alcohol at first visit, timing of first trimester visit, women with adjusted placental-associated plasma protein-A (PAPP-A) < 0.3 MoM, and use of assisted reproductive techniques. Those factors which were associated with the outcome on univariable analysis were taken forward for inclusion in the multivariable regression model.

### Management of missing data

The level of missingness was calculated and reported for all sociodemographic and clinical characteristics. Height was missing in 10.4% of records and imputed for these cases using single imputation according to the median height for ethnicity. All analyses were conducted only in those women with a completed set of characteristics and outcomes, after imputation of missing height.

### Statistical methods

The prevalence of pre-pregnancy obesity was calculated in categories of BMI as defined by the World Health Organization (BMI < 18.5 kg/m^2^: underweight, BMI 18.5–24.9 kg/m^2^: normal weight, BMI 25.0–29.9 kg/m^2^: overweight, BMI 30.0–34.9 kg/m^2^: class I obesity, BMI 35.0–39.9 kg/m^2^: class II obesity, and BMI ≥40.0 kg/m^2^: class III obesity) [12], and compared by reporting year.

The rate of uncomplicated pregnancy was calculated using frequency (n) and percentage (%) and compared among women with obesity and with or without early pregnancy complicating factors for antenatal complication using Chi-squared tests. The maternal socio-demographic characteristics and clinical factors of otherwise-healthy women with obesity were described (mean/standard deviation (SD) or n/% as appropriate) and compared between those with complicated and uncomplicated pregnancy (Chi-squared test, student t-test for parametric data and Kruskal-Wallis H test for non-parametric data). Log-transformation was applied to BMI data due to non-normal distribution.

Multivariable log-binomial regression models were used to estimate the adjusted relative risk (aRR) and 95% confidence intervals (95% CI) of the associations between uncomplicated pregnancy and associated sociodemographic or clinical factors, among women with obesity but no other early pregnancy complicating factors and compared to women without obesity or early pregnancy complicating factors. Potential confounders were identified by presence of a 10% change in the measure of association (crude/adjusted RR) for the exposure before and after introduction of confounders into the model. Where confounders were identified, these were used to adjust the final multivariable model.

Backward stepwise logistic regression was then used, with Akaike’s Information Criteria as the stop rule, to identify factors that were independently associated with uncomplicated pregnancy. The area under the receiver operating characteristic curve (AUROC) was then used to calculate the accuracy of the multivariable model in discriminating between women with complicated and uncomplicated pregnancy.

All analyses were performed using the Statistical Analysis System (SAS) for Windows, version 9.4 (SAS Institute, Cary.NC).

## Results

**Fig. 1:**
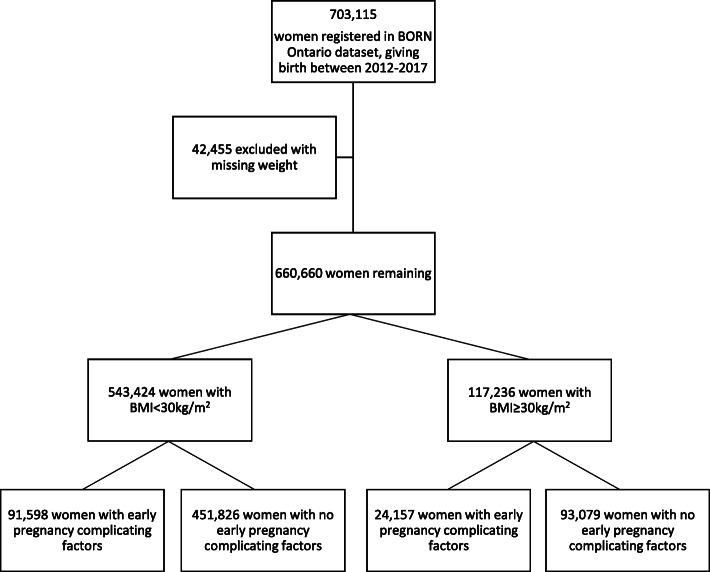
Composition of the cohort population

Between 2012 and 2017, there were 703,115 pregnancies with data recorded in the BORN Ontario registry. Following exclusion of 42,455 women with missing weight (6.0%), the final study population was composed of 660,660 women. Of these, 17.7% (*n* = 117,236) had a recorded BMI ≥ 30 kg/m^2^ (Fig. 1). This is very similar to the rate of obesity reported for women aged 18–34 years, living in Ontario during 2016 (17.4%) [13]. The rate of obesity slowly increased year-on-year, from 17.4% in 2012/13 to 18.1% in 2016/17. Among women with obesity, 59.2% (*n* = 69,383) of women had class I obesity, 25.1% (*n* = 29,386) had class II obesity and 15.8% (*n* = 18,467) of women had class III obesity.

Of women with BMI ≥30 kg/m^2^, 20.6% of women had early pregnancy complicating factors (8.8% had medical risks, 13.5% had early obstetric complicating factors – some had both), compared to 16.5% of women with BMI 18.5–24.9 kg/m^2^ (*p* < 0.001) (Table 2). The reduction in the latter group was mostly in pre-existing medical complicating factors (8.8 to 5.0%; p < 0.001). The rates of pre-existing medical and early obstetric complicating factors for women within each BMI group are presented in Table 2.

**Table 2:** Rate of pre-existing medical or obstetric complicating factors for adverse antenatal outcome, stratified by body mass index group

BMI (kg/m^2^)	Women with early pregnancy complicating factors						Women without early pregnancy complicating factors		Total
	Pre-existing medical complicating factor(s)		Obstetric complicating factor(s)		Pre-existing medical and/or early obstetric factors (subtotal)				
	n	%	n	%	n	%	n	%	n
< 18·5	2124	5·4	5365	13·6	7070	17·9	32,336	82·1	39,406
18·5–24·9	17,290	5·0	42,762	12·4	57,038	16·5	288,387	83·5	345,425
25·0–29·9	9418	5·9	19,882	12·5	27,490	17·3	131,103	82·7	158,593
30·0–34·9	5293	7·6	9193	13·2	13,465	19·4	55,918	80·6	69,383
35·0–39·9	2820	9·6	4042	13·8	6331	21·5	23,055	78·5	29,386
40·0–49·9	1884	11·7	2251	14·0	3754	23·4	12,285	76·6	16,039
50·0+	312	12·9	372	15·3	607	25·0	1821	75·0	2428

The rate of each complicating antenatal diagnosis from the ‘complicated pregnancy’ composite outcome is presented in Table 3 for the women without early pregnancy complicating factors, stratified by BMI group. For comparison, this table has also been reproduced for women with early pregnancy complicating factors in the Additional file 2. The rate of pregnancy-induced hypertension, gestational diabetes, intrauterine fetal death, large-for-gestational-age infant, and development of any pregnancy complication increases with advancing BMI category, both for women with and without early pregnancy complicating factors. The rate of preterm birth was highest for women with a low (< 18.5 kg/m2) or high (≥30 kg/m^2^) BMI (‘U’-shaped curve relationship). The rate of small-for-gestational-age/growth restricted infant and antepartum bleeding decreases with advancing BMI category but other complications have no clear trend.

**Table 3:** Rate of specific antenatal complications in women with no early pregnancy complicating factors, stratified by BMI group

BMI (kg/m^2^)	Pregnancy hypertension		Gestational diabetes		Fetal anomaly		Intrauterine fetal death		IUGR		SGA		LGA		Antepartum bleeding/ abruption		Preterm birth		Premature rupture of membrane		Any antenatal complication		Total
	n	%	n	%	n	%	n	%	n	%	n	%	n	%	n	%	n	%	n	%	n	%	N
< 18·5	489	1·5	897	2·8	241	0·7	74	0·2	1474	4·6	3977	12·3	1110	3·4	283	0·9	1878	5·8	301	0·9	9479	29·3	32,336
18·5–24·9	7512	2·6	11,600	4·0	2201	0·8	587	0·2	7272	2·5	21,224	7·4	20,925	7·3	1942	0·7	13,845	4·8	3218	1·1	78,762	27·3	288,387
25·0–29·9	6194	4·7	9261	7·1	1023	0·8	329	0·3	2648	2·0	7263	5·5	15,670	12·0	866	0·7	6550	5·0	1537	1·2	43,253	33·0	131,103
30·0–34·9	4346	7·8	5484	9·8	431	0·8	175	0·3	984	1·8	2696	4·8	8713	15·6	339	0·6	3086	5·5	659	1·2	21,831	39·0	55,918
35·0–39·9	2384	10·3	2833	12·3	149	0·6	77	0·3	387	1·7	1011	4·4	4502	19·5	132	0·6	1341	5·8	279	1·2	10,275	44·6	23,055
40·0–49·9	1626	13·2	1725	14·0	74	0·6	48	0·4	206	1·7	487	4·0	2596	21·1	85	0·7	8,01	6·5	124	1·0	5956	48·5	12,285
50·0+	224	12·3	226	12·4	12	0·7	5	0·3	24	1·3	84	4·6	369	20·3	10	0·5	114	6·3	13	0·7	826	45·4	1821

*BMI* Body mass index, *IUGR* intrauterine growth restriction (birthweight <3rd centile for gestational age), *SGA* small for gestational age, *LGA* large for gestational age

Of 93,079 women with obesity but no early pregnancy complicating factors for antenatal complication, 54,191 (58.2%) had uncomplicated pregnancies. Of 24,157 women with obesity and other early pregnancy complicating factors, only 10,978 (45.4%) had uncomplicated pregnancies. The relative risk of complicated pregnancy for women with obesity and early pregnancy complicating factors, compared to otherwise healthy women who have obesity was 1.67 (95% CI: 1.63–1.72, *p* < 0.001).

Of women with obesity and no identified early pregnancy complicating factors, women with uncomplicated pregnancies were more likely to be younger, of a lower class of obesity, multiparous, Black or Caucasian ethnicity (than Asian or Other ethnicities), and in the highest two quintiles for neighbourhood income. They were less likely to have a low PAPP-A measurement, be of Asian ethnicity, and have conceived through assisted reproductive technology. On univariable analysis, first language (RR 0.99, 95% CI 0.96–1.01, *p* = 0.28), smoking status (RR 0.99, 95% CI 0.94–1.03, *p* = 0.87), alcohol consumption (RR 1.02, 95% CI 0.98–1.06, *p* = 0.35), and timing of first trimester visit (RR 0.97, 95% CI 0.92–1.02, *p* = 0.27) were not associated with the uncomplicated pregnancy outcome and therefore not included in the multivariable model. Frequency, percentage, crude and adjusted relative risks are presented in Table 4.

**Table 4:** Characteristics associated with uncomplicated pregnancy in women with obesity but no other early pregnancy complicating factors

	All women	Uncomplicated pregnancy (***n*** = 21,776)		Complicated pregnancy (***n*** = 16,279)		Crude RR^**a**^ (95% CI)	Adjusted RR^**a**^ (95% CI)
	N	Mean / n	SD / %	n	%		
**Maternal age (years)**							
Mean (SD)	81,014	30·1	5·1	35·0	4·9	NA	NA
/1-year increase	NA	NA	NA	NA	NA	0·99 (0·98–0.99)	0·99 (0·98–0·99)
**Pre-pregnancy BMI**							
Mean (SD)	81,014	30·9	5·1	35·6	5·1	NA	NA
/unit increase in log (BMI)	NA	NA	NA	NA	NA	0·32 (0·29–0·36)	0·31 (0·27–0·34)
**PAPP-A (MoM)**							
≥ 0·3	37,116	21,332	57·5%	15,784	42·5%	Reference	Reference
< 0·3	939	444	47·3%	495	52·7%	0.82 (0.77–0.88)	0.82 (0.77–0.88)
Missing	42,959	25,446	59·2%	17,513	40·8%	1·03 (1·02–1·04)	1·00 (0·99–1·02)
**Neighbourhood income level**							
Quintile 1 (lowest)	20,844	12,208	58·6%	8636	41·4%	1·00 (0·98–1·02)	0·98 (0·96–0·998)
Quintile 2	16,193	9316	57·5%	6877	42·5%	0·98 (0·96–0·999)	0·97 (0·96–0·99)
Quintile 3	16,390	9615	58·7%	6775	41·3%	Reference	Reference
Quintile 4	17,675	10,275	58·1%	7400	41·9%	0·99 (0·97–1·01)	1·00 (0·98–1·02)
Quintile 5 (highest)	9912	5808	58·6%	4104	41·4%	1.00 (0.98–1.02)	1·01 (0·99–1·04)
**Parity**							
Nulliparous	31,380	17,738	56·5%	13,642	43·5%	Reference	Reference
Multiparous	49,634	29,484	59·4%	20,150	40·6%	1·05 (1·04–1·06)	1·09 (1·07–1·10)
**Ethnicity**							
Caucasian	35,513	20,665	58·2%	14,848	41·8%	Reference	Reference
Asian	6006	3103	51·7%	2903	48·3%	0·89 (0·87–0·91)	0·88 (0·85–0·90)
Black	4676	2850	60·9%	1826	39·1%	1·05 (1·02–1·07)	1·03 (1·01–1·06)
Other	3107	1705	54·9%	1402	45·1%	0·94 (0·91–0·98)	0·93 (0·90–0·96)
Unknown	31,712	18,899	59·6%	12,813	40·4%	1·02 (1·01–1·04)	0.99 (0.98–1.01)
**Conception type**							
In-vitro fertilization	1254	570	45·5%	684	54·5%	0·77 (0·73–0·82)	0·85 (0·80–0·90)
Intrauterine insemination	2027	1022	50·4%	1005	49·6%	0·86 (0·82–0·90)	0·92 (0·88–0·96)
Spontaneous	77,733	45,630	58·7%	32,103	41·3%	Reference	Reference

RR adjusted for all other factors listed in the table

^a^RR < 1 indicates lower chance of uncomplicated pregnancy (i.e., more likely to be complicated) and vice versa

The characteristics predictive of uncomplicated pregnancy in women with obesity but no early pregnancy complicating factors were compared to those predictive for women of healthy weight (BMI 18.5–24.9 kg/m^2^) or overweight (BMI 25.0–29.9 kg/m^2^) and without early pregnancy complicating factors for antenatal complication.

The predictive characteristics for women of healthy weight are summarised in Additional files 3 and 4. In women of healthy weight, PAPP-A < 0.3 MoM was more strongly associated with uncomplicated pregnancy outcome (aRR 0.76, 95% CI 0.73–0.79) compared to women with obesity (aRR 0.82, 95% CI 0.77–0.88). BMI category was confirmed as an effect modifier for the relationship with PAPP-A and uncomplicated pregnancy amongst women without early pregnancy co-morbidities or complications by post hoc Wald test (*p* < 0.0001).

Following stepwise logistic regression, maternal age, BMI, PAPP-A < 0.3 MoM, neighbourhood income quintiles 1 or 2, multiparity, ethnicity, and artificial reproductive techniques remained as independent predictors for the uncomplicated pregnancy outcome in women with obesity but no other early pregnancy complicating factors. The point estimates and 95% confidence intervals for the odds ratios are detailed in Table 5. Using the final prediction model, the AUROC for uncomplicated pregnancy was 0.578.

**Table 5:** Point estimate of the independent odds ratios of predictors for uncomplicated pregnancy in women with obesity but no other early pregnancy complicating factors

Predictor		Adjusted relative risk	95% Confidence Interval
Maternal age (per 1 year of age)		0·96	0·96–0·97
BMI (/ 1 unit of log (BMI))		0·07	0·05–0·09
PAPP-A < 0·3 MoM		0·65	0·57–0·74
Neighbourhood income quintile	Quintile 1	0·95	0·91–0·98
	Quintile 2	0·93	0·89–0·96
Multiparity		1·22	1·18–1·25
Ethnicity	Asian	0·75	0·71–0·79
	Black	1·11	1·04–1·18
	Other	0·84	0·78–0·91
Artificial reproductive therapies	In-vitro fertilization	0·73	0·65–0·82
	Intrauterine insemination	0·84	0·77–0·92

## Discussion

This study aimed to calculate the rate of uncomplicated pregnancy among pregnant women with obesity and to determine the demographic and clinical factors which are associated with such uncomplicated pregnancies.

Through this large prospective cohort study of women with obesity in Ontario, Canada, we have identified that 79.4% of women with obesity had no other early pregnancy complicating factors (obstetric or co-morbidities). Of these, 58.2% had an uncomplicated pregnancy. Uncomplicated pregnancy was significantly more likely (RR: 1.28, 95% CI 1.26–1.30, *p* < 0.001) for women with obesity but no early pregnancy complicating factors compared to women with obesity and early pregnancy factors (45.4%). In women with obesity but without other early pregnancy complicating factors, an uncomplicated pregnancy was more likely if they were younger, with lower BMI, multiparous, black or Caucasian ethnicity or in the highest three quintiles for neighbourhood income; and less likely if the women had a low PAPP-A measurement at prenatal screening, were of Asian ethnicity or had conceived through artificial reproductive techniques. At present, these predictive factors identified through population-level routinely collected data were not strong enough to discriminate between uncomplicated and complicated pregnancy outcomes in this group.

### Comparison with existing literature

The prevalence of uncomplicated pregnancy in this study is very similar to the rates reported in the UK Birthplace cohort, where less than half (45.4%) of women with obesity had either a pre-existing or new obstetric risk factor (e.g., previous Caesarean section, pre-eclampsia) at the time of admission to the maternity unit for birth [5], and the Dutch cohort study (2014), where 55% of women with BMI > 35 kg/m^2^ did not require referral for obstetric care during pregnancy [6].

Where our study differs from these national cohort studies is in the comparison of complications for women with obesity with and without pre-existing complicating factors, and in the study of characteristics associated with an uncomplicated pregnancy outcome. As in Vieira et al’s proof of concept study (2017) uncomplicated pregnancy was independently associated with multiparity and younger age [7]. Many of the predictors in Vieira’s study are not routinely collected, limiting immediate translational potential. We also identified additional predictors in BMI (continuously reported), ethnicity, markers of socioeconomic deprivation, use of fertility treatments and PAPP-A; this information is routinely available for the majority of women giving birth in moderate- or high-income settings.

### Strengths and limitations

The strengths of this study are in the large cohort used from a country with universal access to healthcare, providing an opportunity to identify risk of even rare outcomes, e.g., intrauterine fetal death, and in the methodology to stratify women with obesity according to presence or absence of early pregnancy complications which will inform pathways of prenatal of care. It is important to note the methodological limitations of using a routinely collected dataset, including our choice of using self-reported pre-pregnancy weight where measured weight was not available and the decision to impute height using single imputation for mean height in the ethnic group for the 10.4% of women for whom height was not recorded. It has previously been established that heavier women are more likely to underestimate their weight when self-reporting [14, 15], however self-reported and measured values for BMI are highly correlated [16–18]. Whilst our assumptions may have led to a small proportion of women being placed into the incorrect BMI category, given the large sample size, uncertainty in the presented estimates is expected to be minimal. Finally, we did not have access to data on clinical and biochemical measurements (e.g., systolic blood pressure or parameters of glucose metabolism) which have previously been shown to improve prediction of uncomplicated outcomes in this population [7].

### Implications for practice and policy

In some settings it is routine practice to manage otherwise low-risk nulliparous women in midwifery-led or primary care settings where available, if they choose to do so. The rate of complicated outcomes in the group of women with obesity appears to be similar, it therefore follows that these women with obesity should also have the option of having prenatal care in midwifery-led settings, assuming that appropriate surveillance and timely reflex referral to higher-risk care providers continues for potential complications (e.g., routine screening for gestational diabetes and blood pressure/urine tests for signs of pre-eclampsia).

### Further research

Confidence in risk stratification strategies for this group of women is expected to improve with the aid of decision support tools, incorporating predictive algorithms. The predictive power demonstrated in this study was limited by the absence of clinical, ultrasound and biochemical measurements. Other cohort studies have recognised that these additional factors, not present in routinely-collected registries, augment predictive power [19, 20]. Furthermore, the different performance of biochemical markers for prediction of adverse outcome in pregnant women with obesity has previously also been shown [7, 21]. Further research is required to develop, test and validate a predictive tool, specific to pregnant women with obesity, which incorporates these additional parameters, and to conduct a similar analysis which separately identifies the rate and factors associated with uncomplicated birth amongst women with obesity but otherwise uncomplicated prenatal periods.

## Conclusions

The study demonstrates that over half of women with obesity, but no other pre-existing medical or early obstetric complicating factors proceed through pregnancy without adverse obstetric complication. This is similar to the rate of uncomplicated pregnancy noted in nulliparous women through an international cohort study [22]. Whilst the ability to predict uncomplicated outcomes needs further research, acknowledgement and prediction has the potential to personalise the provision of care to women with obesity, including provision of care in low risk settings for women most likely to have uncomplicated pregnancies, and triaging women at high risk of complications into care settings which are set up to monitor for and offer treatment for these.

## Supplementary Information

**Additional file 1.** STROBE checklist

**Additional file 2: Appendix Table 1**. Rate of specific antenatal complications in women with early pregnancy complicating factors, stratified by BMI group

**Additional file 3: Appendix Table 2**. Characteristics associated with uncomplicated pregnancy in women of healthy weight (BMI 18.5–24.9 kg/m2) but no other early pregnancy complicating factors

**Additional file 4: Appendix Table 3**. Characteristics associated with uncomplicated pregnancy in women who are overweight (BMI 25.0–29.9 kg/m) but no other early pregnancy complicating factors

## Data Availability

The data analysed during this study is held securely at the prescribed registry BORN Ontario. Data sharing regulations prevent this data from being made available publicly due to the personal health information in the datasets. Enquiries regarding BORN data must be directed to BORN Ontario (Science@BORNOntario.ca).

## Abbreviations

AUROC: Area under the receiver operating curve
BMI: Body mass index
BORN: Better Outcomes Registry and Network
CI: Confidence Interval
IUGR: Intrauterine growth restriction
LGA: Large for gestational age
MoM: Multiples of median
PAPP-A: Placenta associated plasma protein A
RR: Relative risk
SD: Standard deviation
SGA: Small for gestational age
STROBE: STrengthening Reporting OBservational studies in Epidemiology
UK: United Kingdom
