# Supplementary material for: Characteristics associated with uncomplicated pregnancies in women with obesity: a population-based cohort study
Source: BMC Pregnancy Childbirth. 2021 Mar 5;21:182. doi: 10.1186/s12884-021-03663-2 (PMC7934497; doi:10.1186/s12884-021-03663-2)
Supplement: Supplementary file 2 — Additional file 2: Appendix Table 1. Rate of specific antenatal complications in women with early pregnancy complicating factors, stratified by BMI group [file 12884_2021_3663_MOESM2_ESM.docx]

Appendix Table 1 - Rate of specific antenatal complications in women with early pregnancy complicating factors, stratified by BMI group

| BMI (kg/m2) | Pregnancy hypertension | | Gestational diabetes | | Fetal anomaly | | Intrauterine fetal death | | IUGR | | SGA | | LGA | | Antepartum bleeding/ abruption | | Preterm birth | | Premature rupture of membrane | | Any antenatal complication | | Total |
| --- | --- | --- | --- | --- | --- | --- | --- | --- | --- | --- | --- | --- | --- | --- | --- | --- | --- | --- | --- | --- | --- | --- | --- |
|  | **n** | **%** | **n** | **%** | **n** | **%** | **n** | **%** | **n** | **%** | **n** | **%** | **n** | **%** | **n** | **%** | **n** | **%** | **n** | **%** | **n** | **%** | **N** |
| <18·5 | 219 | 3.1% | 222 | 3.1% | 85 | 1.2% | 27 | 0.4% | 361 | 5.1% | 890 | 12.6% | 201 | 2.8% | 97 | 1.4% | 1,234 | 17.5% | 52 | 0.7% | 2,814 | 39.8% | 7,070 |
| 18·5-24·9 | 2,879 | 5% | 2494 | 4.4% | 757 | 1.3% | 214 | 0.4% | 1,713 | 3.0% | 4,298 | 7.5% | 3,730 | 6.5% | 631 | 1.1% | 9,068 | 15.9% | 514 | 0.9% | 21,220 | 37.2% | 57,038 |
| 25·0-29·9 | 2,631 | 9.6% | 2129 | 7.7% | 354 | 1.3% | 136 | 0.5% | 662 | 2.4% | 1,602 | 5.8% | 2,976 | 10.8% | 270 | 1.0% | 4,780 | 17.4% | 290 | 1.1% | 12,082 | 44.0% | 27,490 |
| 30·0-34·9 | 1,949 | 14.5% | 1464 | 10.9% | 176 | 1.3% | 84 | 0.6% | 295 | 2.2% | 697 | 5.2% | 1,952 | 14.5% | 132 | 1.0% | 2,491 | 18.5% | 115 | 0.9% | 6,922 | 51.4% | 13,465 |
| 35·0-39·9 | 1,175 | 18.6% | 874 | 13.8% | 88 | 1.4% | 53 | 0.8% | 136 | 2.1% | 299 | 4.7% | 1,108 | 17.5% | 56 | 0.9% | 1,237 | 19.5% | 56 | 0.9% | 3,580 | 56.5% | 6,331 |
| 40·0-49·9 | 927 | 24.7% | 591 | 15.7% | 49 | 1.3% | 26 | 0.7% | 83 | 2.2% | 134 | 3.6% | 792 | 21.1% | 34 | 0.9% | 734 | 19.6% | 32 | 0.9% | 2,301 | 61.3% | 3,754 |
| 50·0+ | 136 | 22.4% | 93 | 15.3% | 11 | 1.8% | 6 | 1.0% | 11 | 1.8% | 26 | 4.3% | 148 | 24.4% | 6 | 1.0% | 117 | 19.3% | 4 | 0.7% | 376 | 61.9% | 607 |
| BMI: Body mass index; IUGR: intrauterine growth restriction (birthweight <3^rd^ centile for gestational age), SGA: small for gestational age; LGA: large for gestational age. | | | | | | | | | | | | | | | | | | | | | | | |
