# Supplementary material for: Characteristics associated with uncomplicated pregnancies in women with obesity: a population-based cohort study
Source: BMC Pregnancy Childbirth. 2021 Mar 5;21:182. doi: 10.1186/s12884-021-03663-2 (PMC7934497; doi:10.1186/s12884-021-03663-2)
Supplement: Supplementary file 3 — Additional file 3: Appendix Table 2. Characteristics associated with uncomplicated pregnancy in women of healthy weight (BMI 18.5–24.9 kg/m2) but no other early pregnancy complicating factors [file 12884_2021_3663_MOESM3_ESM.docx]

Appendix Table 2 - Characteristics associated with uncomplicated pregnancy in women of healthy weight (BMI 18.5-24.9kg/m2) but no other early pregnancy complicating factors

|  | **All women** | **Uncomplicated pregnancy (n=21776)** | | **Complicated pregnancy (n=16279)** | | **Crude RR (95% CI)** | **Adjusted RR (95% CI)** |
| --- | --- | --- | --- | --- | --- | --- | --- |
|  | N | Mean / n | SD / % | n | % |  |  |
| **Maternal age (years)** | | | | | | | |
| Mean (SD) | 245,340 | 30.2 | 5.0 | 30.5 | 5.2 |  |  |
| /1 year increase |  |  |  |  |  | 0.997 (0.996-0.997) | 0.995 (0.994-0.995) |
| **Pre-pregnancy BMI** | | | | | | | |
| Mean (SD) | 245,340 | 21.8 | 1.7 | 21.9 | 1.8 |  |  |
| /unit increase in log(BMI) |  |  |  |  |  | 0.63 (0.59-0.67) | 0.60 (0.56-0.64) |
| **PAPP-A (MoM)** | | | | | | | |
| ≥0·3 | 121,364 | 88,366 | 72.8% | 32,998 | 27.2% | Reference | Reference |
| <0·3 | 2,059 | 1,129 | 54.8% | 930 | 45.2% | 0.75 (0.72-0.78) | 0.76 (0.73-0.79) |
| Missing | 121,917 | 89,547 | 73.4% | 32,370 | 26.6% | 1.01 (1.00-1.01) | 1.00 (0.996-1.01) |
| **Neighbourhood income level** | | | | | | | |
| quintile 1 (lowest) | 50,735 | 36,207 | 71.4% | 14,528 | 28.6% | 0.98 (0.97-0.99) | 0.98 (0.97-0.99) |
| quintile 2 | 42,598 | 31,005 | 72.8% | 11,593 | 27.2% | 1 (0.99-1.01) | 1 (0.99-1.00) |
| quintile 3 | 47,544 | 34,696 | 73.0% | 12,848 | 27.0% | Reference | Reference |
| quintile 4 | 60,515 | 44,410 | 73.4% | 16,105 | 26.6% | 1.01 (1.00-1.01) | 1.01 (1.00-1.01) |
| quintile 5 (highest) | 43,948 | 32,724 | 74.5% | 11,224 | 25.5% | 1.02 (1.01-1.03) | 1.02 (1.01-1.03) |
| **Parity** | | | | | | | |
| Nulliparous | 116,826 | 82,332 | 70.5% | 34,494 | 29.5% | Reference | Reference |
| Multiparous | 128,514 | 96,710 | 75.3% | 31,804 | 24.7% | 1.07 (1.06-1.07) | 1.08 (1.08-1.09) |
| **Race** | | | | | | | |
| Caucasian | 98,731 | 73,812 | 74.8% | 24,919 | 25.2% | Reference | Reference |
| Asian | 48,221 | 32,926 | 68.3% | 15,295 | 31.7% | 0.91 (0.91-0.92) | 0.92 (0.91-0.92) |
| Black | 7,337 | 5,308 | 72.3% | 2,029 | 27.7% | 0.97 (0.95-0.98) | 0.97 (0.95-0.98) |
| Other | 8,162 | 5,879 | 72.0% | 2,283 | 28.0% | 0.96 (0.95-0.98) | 0.97 (0.95-0.98) |
| Unknown | 82,889 | 61,117 | 73.7% | 21,772 | 26.3% | 0.99 (0.98-0.99) | 0.97 (0.97-0.98) |
| **Conception type** | | | | | | | |
| IVF/IVF ICSI | 3,959 | 2,544 | 64.3% | 1,415 | 35.7% | 0.88 (0.86-0.90) | 0.91 (0.89-0.93) |
| IUI or other | 3,485 | 2,407 | 69.1% | 1,078 | 30.9% | 0.94 (0.92-0.97) | 0.97 (0.95-0.99) |
| Spontaneous conception | 237,896 | 174,091 | 73.2% | 63,805 | 26.8% | Reference | Reference |
