# Supplementary material for: Characteristics associated with uncomplicated pregnancies in women with obesity: a population-based cohort study
Source: BMC Pregnancy Childbirth. 2021 Mar 5;21:182. doi: 10.1186/s12884-021-03663-2 (PMC7934497; doi:10.1186/s12884-021-03663-2)
Supplement: Supplementary file 4 — Additional file 4: Appendix Table 3. Characteristics associated with uncomplicated pregnancy in women who are overweight (BMI 25.0–29.9 kg/m) but no other early pregnancy complicating factors [file 12884_2021_3663_MOESM4_ESM.docx]

Appendix Table 3- Characteristics associated with uncomplicated pregnancy in women who are overweight (BMI 25.0-29.9kg/m) but no other early pregnancy complicating factors

|  | **All women** | **Uncomplicated pregnancy (n=21776)** | | **Complicated pregnancy (n=16279)** | | **Crude RR (95% CI)** | **Adjusted RR (95% CI)** |
| --- | --- | --- | --- | --- | --- | --- | --- |
|  | N | Mean / n | SD / % | n | % |  |  |
| **Maternal age (years)** | | | | | | | |
| Mean (SD) | 113,066 | 30.4 | 5.0 | 31.0 | 5.2 |  |  |
| /1 year increase |  |  |  |  |  | 0.993 (0.993-0.994) | 0.99 (0.99-0.99) |
| **Pre-pregnancy BMI** | | | | | | | |
| Mean (SD) | 113,066 | 27.1 | 1.4 | 27.2 | 1.4 |  |  |
| /unit increase in log(BMI) |  |  |  |  |  | 0.44 (0.43-0.45) | 0.32 (0.27-0.38) |
| **PAPP-A (MoM)** | | | | | | | |
| ≥0·3 | 54,334 | 36,142 | 66.5% | 18,192 | 33.5% | Reference | Reference |
| <0·3 | 1,235 | 655 | 53.0% | 580 | 47.0% | 0.8 (0.76-0.84) | 0.81 (0.77-0.86) |
| Missing | 57,497 | 39,118 | 68.0% | 18,379 | 32.0% | 1.02 (1.01-1.03) | 1.01 (1.00-1.02) |
| **Neighbourhood income level** | | | | | | | |
| quintile 1 (lowest) | 26,210 | 17,469 | 66.7% | 8,741 | 33.3% | 0.99 (0.98-1.01) | 0.99 (0.98-1.00) |
| quintile 2 | 20,594 | 13,802 | 67.0% | 6,792 | 33.0% | 1.00 (0.99-1.01) | 0.99 (0.98-1.01) |
| quintile 3 | 22,580 | 15,151 | 67.1% | 7,429 | 32.9% | Reference | Reference |
| quintile 4 | 26,631 | 17,942 | 67.4% | 8,689 | 32.6% | 1.00 (0.99-1.02) | 1.01 (1.00-1.02) |
| quintile 5 (highest) | 17,051 | 11,551 | 67.7% | 5,500 | 32.3% | 1.01 (1.00-1.02) | 1.02 (1.00-1.03) |
| **Parity** | | | | | | | |
| Nulliparous | 46,127 | 29,996 | 65.0% | 16,131 | 35.0% | Reference | Reference |
| Multiparous | 66,939 | 45,919 | 68.6% | 21,020 | 31.4% | 1.05 (1.05-1.06) | 1.08 (1.07-1.09) |
| **Race** | | | | | | | |
| Caucasian | 45,725 | 31,321 | 68.5% | 14,404 | 31.5% | Reference | Reference |
| Asian | 16,683 | 10,073 | 60.4% | 6,610 | 39.6% | 0.88 (0.87-0.89) | 0.89 (0.88-0.90) |
| Black | 5,541 | 3,773 | 68.1% | 1,768 | 31.9% | 0.99 (0.98-1.01) | 0.99 (0.97-1.01) |
| Other | 4,473 | 2,934 | 65.6% | 1,539 | 34.4% | 0.96 (0.94-0.98) | 0.95 (0.93-0.97) |
| Unknown | 40,644 | 27,814 | 68.4% | 12,830 | 31.6% | 1.00 (0.99-1.01) | 0.97 (0.96-0.99) |
| **Conception type** | | | | | | | |
| IVF/IVF ICSI | 1,831 | 1,038 | 56.7% | 793 | 43.3% | 0.84 (0.81-0.88) | 0.9 (0.86-0.93) |
| IUI or other | 1,940 | 1,201 | 61.9% | 739 | 38.1% | 0.92 (0.89-0.95) | 0.96 (0.93-0.99) |
| Spontaneous conception | 109,295 | 73,676 | 67.4% | 35,619 | 32.6% | Reference | Reference |
